# Multi-endpoint effects of derelict tubular mussel plastic nets on *Tigriopus fulvus*

**DOI:** 10.1007/s11356-022-21569-0

**Published:** 2022-06-29

**Authors:** Isabella Parlapiano, Francesca Biandolino, Asia Grattagliano, Andrea Ruscito, Giusy Lofrano, Giovanni Libralato, Marco Trifuoggi, Luisa Albarano, Ermelinda Prato

**Affiliations:** 1 National Research Council, Water Research Institute (IRSA-CNR), Via Roma, 3, 74123 Taranto, Italy; 2 Department of Chemical Sciences and Technologies, University of Rome “Tor Vergata”, Via della Ricerca Scientifica, 1 – 00133 Rome, Italy; 3 Centro Servizi Metrologici E Tecnologici Avanzati (CeSMA), University of Naples Federico II, Via Vicinale Cupa Cintia 26, 80126 Naples, Italy; 4 Dipartimento di Scienze Motorie, Umane e della Salute, Università degli Studi di Roma Foro Italico, Piazza Lauro De Bosis, 15, 00135 Rome, Italy; 5 Department of Biology, University of Naples Federico II, Via Vicinale Cupa Cintia 26, 80126 Naples, Italy; 6 Department of Chemical Sciences, University of Naples Federico II, Via Vicinale Cupa Cintia 26, 80126 Naples, Italy

**Keywords:** Microplastic waste, Leachates, Acute and sub-chronic toxicity, Seawater, *Tigriopus fulvus*

## Abstract

Microplastic debris from direct and indirect human activities is considered a major threat to the marine biodiversity mainly due to its abundance, durability, persistence, and ability to accumulate contaminants from the environment. Derelict tubular plastic nets of various colours (blue (BN), yellow (YN), green (GN), pink (PN), and white (WN) net), used to distinguish mussel farming owners, were collected by scuba-dive from the Mar Piccolo of Taranto (Ionian Sea). All nets were made of polypropylene. Investigations looked for potential acute (mortality) and sub-chronic (mortality, larval development and moult release number, and adult percentage after 5–9 days) effects on *Tigriopus fulvus* nauplii considering both whole plastics (microplastic (MP), 50 mg/L) and leachates (12.5–100%). Acute test determined a median lethal concentration (LC50) only for BN for both MPs (107 mg/L) and leachates (50.1%). The prolonged exposure (5 days) to microplastics did not affect the *T. fulvus* survival. After 9 days, YN and BN decreased of approximately 100% larval development.

**Graphical abstract:**

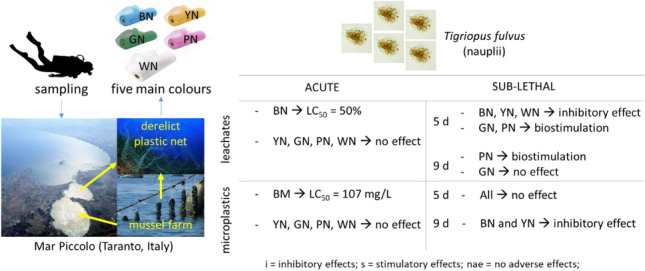

**Supplementary Information:**

The online version contains supplementary material available at 10.1007/s11356-022-21569-0.

## Introduction

Every year, millions of tons of plastic waste is estimated to end up in the oceans, making plastic pollution one of the fastest growing threats to marine ecosystems (Manfra et al. 2021). In 2018, world plastics production was of approximately 359 million tons (PE 2021) and it has been estimated to reach 1800 million tons in 2050 (UNEP 2016). Sea-based plastic waste includes mainly derelict fishing gears (DFGs), such as nets, lines, traps, and other recreational and fishing equipment lost, abandoned, or intentionally discarded at sea (Sheavly 2007). The number of DFGs is increasing in the ocean (Angiolillo et al. 2015, Bilkovic et al. 2014, Macfadyen et al. 2009, Morishige and McElwee 2012, Wilcox et al. 2015) and considering that the aquaculture activities are expected to expand (FAO 2020), the produced waste will proportionally increase, if no action will be undertaken. There is a rising concern about fishing and shellfish industry, since most aquaculture equipment is made mainly of plastic materials, presenting low biodegradability (Andrady 2015; Manfra et al. 2021). In Europe, Italy is the second main producer of sea-farmed mussels after Spain with approximately 64,000 ton/year and is one of the largest European markets for shellfish with an average turnover of 120,000 ton/year (EUMOFA 2019). Sea-based sources, like fishery activities, can contribute up to 60–90% of the total amount of plastic litter (Abu-Hilal and Al-Najjar 2009; Amuda et al. 2017; Angiolillo et al. 2015; Bauer et al. 2008; FAO 2020; Oliveira et al. 2015; Watters et al. 2010), depending on the extent of these activities in the different geographical areas as well as on the relative regulatory policies (Pham et al. 2014). In general, the plastic loads as microplastic (MP) in polluted sea water are within the μg/L range with values up to 0.08 and 0.3 mg/L (Beiras et al. 2018; Lusher et al. 2013). Currently, the adverse effects of plastic use in aquaculture (Khosravi-Katuli et al. 2017) are not limited to primary plastic materials and their incorrect disposal, but they are mainly derived from the generated debris (Avio et al. 2015). The plastics net for mussel growing can be exposed to the mechanical action of waves, scraping, temperature fluctuations, and direct ultraviolet (UV) light degradation contributing to the general embrittlement and fragmentation of macro-plastics to micro- and nano-plastics. They can be readily ingested by a wide range of organisms possessing different feeding strategies and belonging to different trophic levels (Cole et al. 2013; Rochman et al. 2013; Van Cauwenberghe et al. 2015), including many species intended for human consumption, such as bivalve, crustacean (Costa et al. 2020), and fish (Hara et al. 2020; Li et al. 2018; Phuong et al. 2018; Rochman et al. 2015, Van Cauwenberghe and Janssen 2014). Microplastics have been reported as potential active or passive carriers of chemicals including plasticizers, antioxidants, UV stabilizers, colourants, and flame-retardants (Hahladakis et al. 2018) (i.e. active), or adsorbed from the surrounding environment (i.e. passive) such as alkylphenols, phthalate esters, halogenated diphenyl ethers, biphenyl, organochlorine pesticides (Besseling et al. 2013; Rochman et al. 2013, 2014), and heavy metals (Brennecke et al. 2016; Kedzierski et al. 2018; Rochman et al. 2014; Xie et al. 2022) up to several orders of magnitude higher compared to seawater. The ingestion of microplastics can cause a variety of adverse effects ranging from increased mortality (Mazurais et al. 2015), feeding, and fecundity (Yu et al. 2020), clogging/blockage of the digestive tract (de (de Sá et al. 2015), structural and functional intestinal alteration (Pedà et al. 2016), hepatic stress (Rochman et al. 2013), reduction in feeding activity (Besseling et al. 2013; Wright et al. 2013), increased intracellular levels of reactive oxygen species, and changed antioxidant-related gene expression (Choi et al. 2020). Additionally, other authors evidenced that MPs could increase the production of reactive oxygen species (Paul-Pont et al. 2016), alter the gametogenesis (Sussarellu et al. 2016), delay the development reducing fecundity and retarding moulting (Au et al. 2015; Jeong et al. 2017; Lee et al. 2013), considering also the interactions of plastics with additives (Li et al. 2020), and hindering the swimming activity like for tire wear particles (Yang et al. 2022). Several studies suggested that the observed toxicity effects can result from both the chemicals associated with plastics and the plastic material itself (i.e. physical effect) (Avio et al. 2015; Browne et al. 2013; Paul-Pont et al. 2016; Rochman et al. 2013, 2014). For example, Kedzierski et al. (2018) evidenced that aged plastics can rapidly release estrogenic compounds in the marine environment, while Cole et al. (2019) stated that *Calanus finmarchicus* exposed to nylon MPs moulted significantly earlier than copepods in the control treatment. Other authors reported the toxic effects of leachates obtained from MPs. Leachates strongly impaired *Prochlorococcus* in vitro growth and photosynthetic capacity resulting in genome-wide transcriptional changes (Tetu et al. 2019). Similarly, leachates produced adverse effects on the microalgae *Raphidocelis subcapitata* (freshwater), *Skeletonema costatum* (marine) (Capolupo et al. 2020) as well as on mussels (*Mytilus galloprovincialis* and *Perna perna*) and sea urchins on a multi-endpoint basis (Beiras et al. 2019), including the lysosomal membrane stability, gamete fertilization inhibition, embryonic development, and larvae motility and survival (Capolupo et al. 2020; Gandara et al. 2016; Nobre et al. 2015; Oliviero et al. 2019). Li et al. (2016) reported that plastic leachates reduced the survival and settlement of nauplii barnacle *Amphibalanus amphitrite.*

According to the Mediterranean Association of Fish Farmers, plastic litter in the sea was up to 400 tonnes in 2017, and 5% can be originated by aquaculture activities (personnel communication). Fortibuoni et al. (2021) evidenced that in the Adriatic Sea, the densities of litter from aquaculture consisted almost exclusively of mussel nets with a median density of 22 items/100 m reaching extreme values between 295 and 795 items/100 m in four beaches, while Kane et al. (2020) found up to 1.9 million pieces per square metre (i.e. particles size > 63 μm) in the Tyrrhenian Sea. Historically, the Mar Piccolo of Taranto (Italy) is characterized by an intensive mussel farming (i.e. plastic ropes and tubular plastic nets suspended over poles and wooden structures) with an estimated production of about 40,000 tons/year (Giordano et al. 2019). Mussel farmers used esparto grass ropes for juvenile recruitment and farming purposes since the 1970s when tubular plastic nets were firstly introduced. Thus, some areas of the Mar Piccolo of Taranto can suffer from approximately 50 years of periodical mussel net discharge where they stratified in part (i.e. scuba divers personnel communication), but no census exists about. Several years ago, farmers started to use tubular plastic nets of different colours to identify their own production area. Therefore, the bottom area surrounding the mussel farming is characterized by an uncounted stratified amount of DFGs, especially derelict plastic tubular nets, of at least five main colours (blue (BN), yellow (YN), green (GN), pink (PN), and white (WN) net).

The aim of our research was to investigate the potential acute (mortality) and sub-chronic (mortality, larval development and moult release number, and adult percentage after 5–9 days) toxicity of derelict plastic tubular nets in the microplastics form using the marine copepod *Tigriopus fulvu*s as a reference biological model being a primary consumer living at the sediment–water interface (Faraponova et al. 2005, 2016; Mariani et al. 2006; Prato et al. 2011, 2015, 2012, 2013, 2020; Tornambè et al. 2012). Derelict plastic tubular nets were analysed considering five main colours (BN, YN, GN, PN, and WN) to investigate any potential relation between plastic colour and potential observed toxicity.

## Materials and methods

### Plastic net sampling and MP generation

Mussel derelict tubular nets were randomly sampled by a scuba diver from the seabed in an historical and intensive area of mussel production located at the second inlet of Mar Piccolo of Taranto (40° 49′ 37′′N; 17° 31′49′′E) (Southern Italy, Ionian Sea). Sampling included nets of five colours (BN, YN, GN, PN, and WN). Nets were carefully washed on site after sampling to remove any fouling.

Mussel nets were fragmented in an Ultra-Turrax IKAWerkeal (Staufen, Germany). Microplastic particles were selected by sieving through a standard series of sieves (ASTM, model V3SF #635V3SH #400) with increasingly smaller mesh to obtain particles with a size between 20 and 38 µm and stored in glass flasks at room temperature in the dark.

### Polymer identification and MPs characterization

Net polymer identification was performed via attenuated total reflection-Fourier transform infrared (ATR-FTIR) spectroscopy analysis providing information about different functional groups on the polymeric structure. A Nicolet™ iS50 FTIR Spectrometer (Thermo Fischer Scientific, Rome, Italy) equipped with an ATR tool was used to collect spectra in absorbance mode from 4000 to 400 cm^−1^ with a data spacing of 0.482 cm^−1^. Thirty-two scans/sample was used with nominal resolution of 4 cm^−1^ and apodization Beer-Norton strong. A scan of background was run before each measurement. The diamond crystal of ATR tool was cleaned with a mixture of 2/3 (v/v) of ethanol and 1/3 (v/v) of water between every measurement. After this preparation, each plastic sample was put into contact with the diamond crystal and was run the scan of absorption band. The peaks were identified by a spectrometer software, and they were assigned to a spectrum search in commercial and customized polymer libraries.

The size of particles was checked using a dynamic light scattering (DLS) measurement at 90° with a BI‐200SM Goniometer (Brookhaven Instruments Co., Holtsville, NY, USA) equipped with a solid-state laser source at 532 nm and a BI‐9000AT correlation board. Measurements were conducted at 25 °C by transferring 1 mL of stock solution (1 mg/L of MPs, resuspended in Artificial Sea Water IO® (ASW) 37‰) to a square cuvette for DLS analysis, for size characterization, and in distilled water for measuring the effective surface charge. Autocorrelation functions g2 (q, t) of the scattered intensity were analysed by the CONTIN algorithm placed into the software of the instrument.

Polydispersity index (PDI, dimensionless) and zeta (ζ-) potential (mV) were measured as key parameters describing MPs behaviour. Measurements were carried out in triplicate and data was analysed with the dynamic light scattering (DLS) surface zeta potential electrode.

All the measurements were carried out at the start of the test and repeated after 48 h to detect any agglomerates in ASW over time.

### MPs treatment and leachate preparation and chemical analysis

An aliquot of about 0.25 g of MPs per each colour was weighed into Teflon sample tubes. The procedure to extract the elements of interest included the use of aqua regia solution HNO_3_: HCl (1:3) (i.e. 20 mL of 20% aqua regia solution were added to each sample). Extraction was carried out in a microwave for 2 h at 110 °C. Finally, digests were transferred to clean Teflon sample tubes for the subsequent analysis.

The leaching procedure was carried out according to the CEN (2002) with some modifications. To increase the surface area and facilitate leaching, mussels’ nets were cut with clean stainless-steel scissors in about 0.5 mm length pieces and placed in glass beakers. Artificial seawater (ASW) Instant Ocean® (*pH* = 8) was used for leaching (100 g plastic material/L).

Beakers were placed on a horizontal shaker table and the speed was set to 90 rpm allowing the plastic pieces to move freely in the water. Shaking was performed for 24 h at 20 ± 2 °C in the dark. Leachates were obtained filtrating the suspension with Whatman GF/C glass microfiber filters (1.2 µm pore size) to remove larger particles. Filtrated leachates were transferred to a clean tube and stored at 4 °C and used as stock solution for the preparation of serial dilutions.

Samples were analysed with inductively coupled plasma with mass spectrometry (ICP-MS, Aurora M90, Bruker, USA) quantifying Al, Sb, As, Ba, Be, B, Cd, Co, Cr, Fe, Mn, Hg, Mo, Ni, Pb, Cu, Se, V, and Zn. Prior to the analysis, samples were filtered using 0.45 μm regenerated cellulose membrane filters and acidified with 3% v/v HNO_3_ (i.e. only for leachates). The quantitative analysis was performed using an external calibration curve built with five concentrations for each of the analysed elements using multi-element standard solutions for ICP TraceCERT® in 5% nitric acid (Sigma-Aldrich, Milan, Italy) and ultrapure deionized water with conductivity < 0.06 μS/cm. The limit of detection (LOD) and limit of quantification (LOQ) for each metal are reported in Table S1.

### Toxicity tests

Bioassay was performed by using *Tigriopus fulvus* according to the methods described by Faraponova et al. (2016). Individuals were obtained from a massive culture maintained in the CNR — IRSA laboratories of Taranto. Copepods were cultured in filtered (0.45 μm) seawater (38 PSU), in a temperature-controlled room at 20 ± 2 °C under a 16 h light:8 h dark cycle (500–1200 lx) using a mix of Tetramarin ® (fish food) and the microalgae *Tetraselmis suecica* and *Isochrysis galbana* as food source (UNICHIM 2014). CuCl_2_ was employed as a reference toxicant (positive control) to ensure the validity of test. The stock solution of the toxicant (100 mg/L of Cu^+2^) was prepared with ultra-pure water. Seven different test solutions in a geometric concentration series with a factor of two (0.015, 0.03, 0.06, 0.12, 0.25, 0.50, 1.00 mg/L of Cu^2+^) were tested. All tests were carried out on new-borns (nauplii ≤ 24 h-old) of *T. fulvus* released from ovigerous females selected 24 h prior the test and transferred on 80 μm-mesh plankton net fixed to a Plexiglas tube, with food supply (*T. suecica* and *I. galbana* at, respectively, 1.5 × 10^5^ and 3.0 × 10^5^ cells/mL). All toxicity tests were carried out in triplicate and test conditions were the same as described above for copepod culture. Acute and sub-chronic assays testing both microplastics and leachates were repeated three times. Stock solutions (1000 mg/L) were prepared in artificial seawater (ASW) plus Tween20 surfactant to obtain a better microplastics dispersion. The surfactant was added at a concentration (3 µl/L) that did not affect nauplii (Beiras et al. 2018).

Acute toxicity test of MPs included the following concentrations: 50–75–100–125–150 mg/L. During the second screening phase, *T. fulvus* larvae were exposed to 12.5–25–50–75–100% of leachates. Three replicates per dilution were considered and fifteen negative controls (ASW plus Tween20) were carried out. For sub-chronic exposure, based on the results of acute tests, three concentration of MPs (50–100–150 mg/L) and of leachates (25–50–100%) were chosen for all plastics. In the case of BN, the experimental design included the following concentrations: 12.5–25.0–50.0 mg/L for MPs and 3.12–6.25–12.5% for leachates. Test solutions were prepared immediately prior to toxicity testing. For acute tests, pH, temperature, oxygen, and salinity values were measured before and after exposure, for sub-chronic tests were measured before each water change. The toxicity conditions of acute and sub-chronic test are summarized in Table S2.

#### Acute test

Acute toxicity tests (mortality) were performed including both MPs and leachates with ≤ 24 h-old nauplii according to the UNICHIM (2014). Thirty test organisms divided into three groups (replicates) were exposed to each concentration and to the negative control with only ASW for 48 h. Test organisms were randomly selected and transferred in 12-well tissue culture plates (flat bottom, low evaporation) with 3 mL of test solution per chamber. At the end of the exposure, the lethality was assessed counting dead organisms under a stereomicroscope (Leica S9, Leica Microsystems, Milan, Italy). Organisms were considered dead when they were unable to move any external appendage or any internal member for at least 20 s, after gentle stimulation of well solution.

#### Sub-chronic test

Semi-static renewal toxicity tests to evaluate the larval development (i.e. from nauplii to copepodite and from copepodite to adults) and moults release were conducted with newly hatched nauplii (≤ 24 h old) of *T. fulvus* exposed for 9 days to concentrations of MPs and leachates looking for mortality, naupliar moults number and copepodites percentage after 5 days, and copepodite moults number and adults’ percentage after 9 days. Ten nauplii (≤ 24 h-old) of *T. fulvus* were individually introduced into each chamber of 24-well plates containing 1 mL of test solution. ASW without plastics was used as negative control. Nauplii were fed from the beginning of experiment. Test solutions/controls, with food supply (10^5^ cells/mL of *T. suecica*), were renewed after 48 h, transferring the copepods into new well plates containing fresh treatment testing solution and food. Naupliar and copepodite moults were counted on day 5 and 9, respectively. To determine moults number, a drop of Lugol solution was placed into each well, making easier the identification of the exoskeletons under the microscope. Results were reported as the mean number of moults and expressed as moults percentage reduction/increase at different microplastic and leachate concentrations compared to the control. At the same time, the developmental stage was observed to calculate the percentage of individuals that reached the copepodite stage on the day 5 and the adult stage on day 9.

### Statistical analysis

Data are presented as means and standard deviation (S.D., *n* = 3). Acute tests were considered valid if the nauplii mortality in the negative control was ≤ 10%. The 96-h LC50 values and their 95% confidence intervals (CIs) were calculated using linear regression models (e.g. log-logistic or Probit). Data were analysed for normality and homogeneity of variances using Kolmogorov–Smirnov and Bartelett’s tests. When both assumptions were met, data were examined by one-way analysis of variance (ANOVA). If significant differences (*p* < 0.05) were found by the ANOVA, the Tukey’s multiple comparison test was used to discriminate between the means. When requirements for normality and homogeneity were not met, the non-parametric Kruskal–Wallis test on ranks was applied (*p* ≤ 0.05). All statistical analyses were conducted using Statgraphics software and package software Past3 (version 1.0).

## Results

### Plastic composition and size characterization of MPs

At time *T* = 0, the instrument identified a three modal size distribution in ASW with a greater microscale aggregate in the range of approximately 1020–1234 nm and a smaller cluster of microscale aggregates (approximately 177–235 nm). Only green net (GN) showed a bimodal size distribution with a greater aggregate in a range of approximately 925 nm and a smaller microscale aggregate of approximately 533 nm. Microplastics behaviour changed with time increasing their size from *T* = 0 to *T* = 48 h (Table S2). The FT-IR spectra revealed that the five mussel nets presented the same spectral bands, thus a similar chemical composition. In Fig. S1 (Supplementary Materials), the magnified section shows that the IR fingerprint region (from 2000 to 450 cm^−1^) established that all nets are made of polypropylene (PP).

Dynamic light scattering measurements revealed the presence of plastic particles < 1.2 μm (hydrodynamic diameter) in each suspension (Table S3). All polypropylene (PP) mussel nets MPs showed a negative ζ-potential (Table S4).

### Metal contamination

**Fig. 1:**
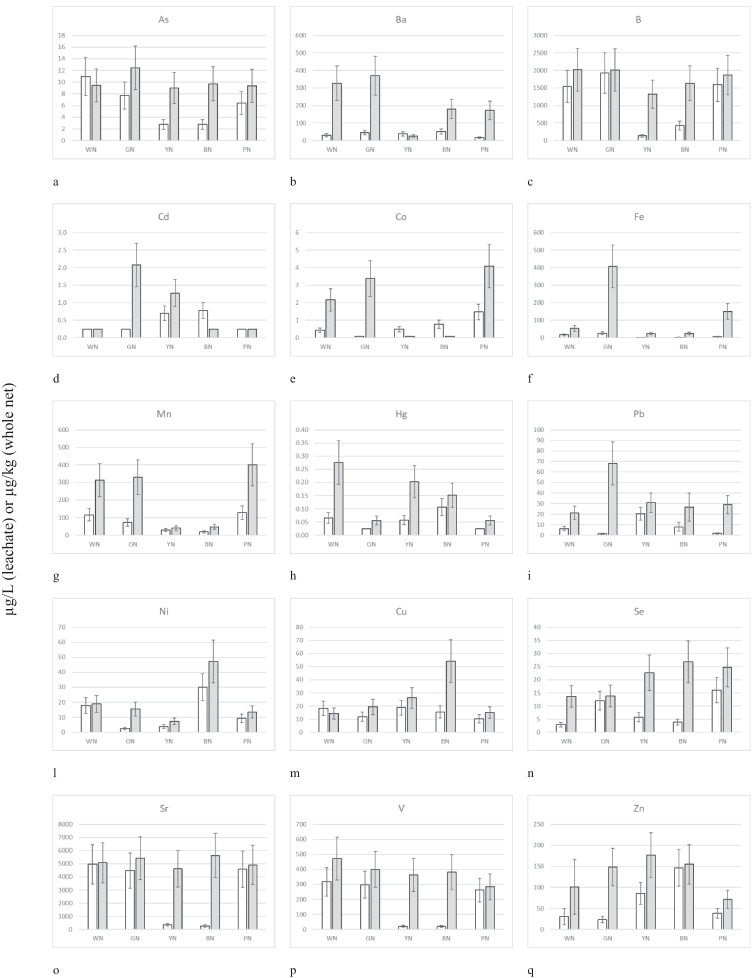
Metalloid (As, B, and Se) and metals (Ba, Cd, Co, Fe, Mn, Hg, Pb, Ni, Cu, Sr, V, and Zn) found in leachate (white bar) and whole plastic (grey bar) nets of various colours (WN, white net; GN, green net; YN, yellow net; BN, black net; PN, pink net); mean values (*n* = 3) ± standard errors; bars without standard errors indicate values below the relative LOD (i.e. half of LOD value)

Metalloid and metal concentrations in microplastic mussel net and the relative leachates were summarized in Fig. 1a–q. A first data overview suggested that plastic nets and the relative leachates did not always contain a comparable number of metalloids and metals. The elemental analysis did not evidence specific critical values. Leachates’ characteristics are in line with the threshold limit values for treated discharges in surface water (Italian Decree 152/2006). Leachates from YN presented the lowest levels of Ba, Co, Fe, Mn, and Hg, like BN for Co, Fe, Mn, Pb, and Se. The highest values were greatly scattered between all colours according to the single monitored element. The content of As, B, Se, Sr, V, and Zn was comparable between whole plastic net samples, while the relative content in leachates presented significant fluctuations according to net colours and the specific element.

**Fig. 2:**
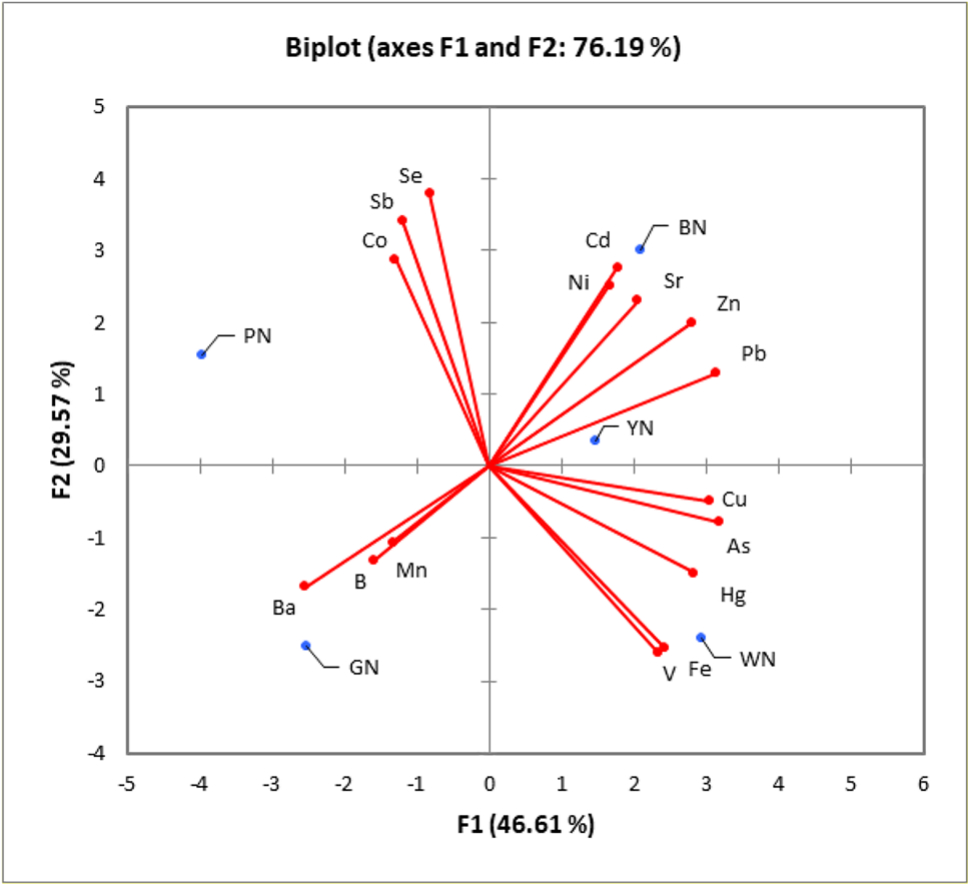
Biplot summarizing the principal component analysis of elements versus coloured plastic samples

A biplot summarizing the PCA results on chemical data for coloured plastic samples was reported in Fig. 2. The first two principal components (F) accounted for 46.61% (F1) and 29.57% (F2) of the variation, respectively. Thus 76.19% of the variation can be depicted by a two-axis ordination diagram. The biplot regarding component loadings suggested that the first component (F1) scores are influenced by the values of As, Fe, Hg, Pb, Cu, Sr, V, and Zn with positive loadings on the first axis, except for Ba. The second component (F2) was mainly influenced by Sb, Cd, Co, Ni, and Se concentrations. The third component identified the presence of B, while the fourth one the presence of Mn. The ordination plot of component scores in Fig. 2 in the F1-F2 biplot evidenced that only BN and YN presented similar characteristics (1st quadrant), while PN, GN, and WN were singly visualized in the 2nd, 3rd, and 4th quadrant, respectively.

### Acute toxicity test

In all acute toxicity tests, the survival of negative controls was > 98%. The reference toxicant LC50 was equal to 0.17 mg/L (0.14–0.22 mg/L) being comparable to (Faraponova et al. 2016; Prato et al. 2015, 2012) (0.18–0.14 mg/L). The median lethal concentration for *T. fulvus* exposed to MPs and leachates was calculable only for BN. In all other samples, nauplii did not show any significant effect compared to negative controls. The LC50 for waste BN nets were 107.1 mg/L (97.4–117.7 mg/L) for MPs and 50.1% (39.3–64.3%) for leachates; copepods mortality showed significant differences compared to the control starting from 75.0 mg/L of MPs and 25% of leachate dilution (*p* < 0.05), respectively.

### Sub-chronic test

**Fig. 3:**
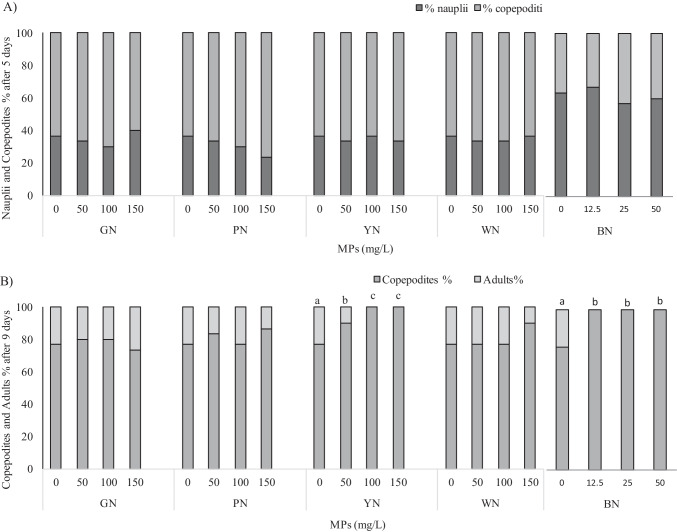
Larval development of *T. fulvus* exposed to different concentrations of GN, PN, YN, WN, and BN microplastics (MPs); bars represent the percentage (%) of nauplii/copepodites after 5 days (**A**) and copepodites/adults after 9 days (**B**). Data with different letters are significantly different (*p* < 0.05, Tukey’s test); no letters indicated that results within the specific treatment/net colour are not significantly different

No lethal effects at any tested concentration were observed for all tested MPs and leachates. In the negative control after 5 days of nauplii exposure (< 24 h-old), the mean number of naupliar moults was 4.8 ± 0.76, while after 9 days was 4.0 ± 0.69 (Fig. 3A and B).

Most of the tested microplastics did not affect larval development of *T. fulvus*: no significant inhibitory or stimulatory effects on naupliar moults release and on larval development from naupliar to copepodite stage was detected after 5 days of exposure to all tested MPs (Fig. 3A).

After 9 days, inhibitory effects on moults release and on the development from copepodite stage to adult stage were observed only in copepods exposed to BN and YN MPs (Fig. 3B).

BN MPs induced a higher inhibitory effect than YN ones. BN MPs showed a percentage reduction of total copepodite moults compared to the control (*p* < 0.05) of 20.8% at 12.5 mg/L and 26.7% at 50 mg/L, while YN MPs showed a reduction of copepodite moults of 9.2% at the lowest concentration, and both at 100 and 150 mg/L the reduction percentage was 19.2% (*p* < 0.05).

**Fig. 4:**
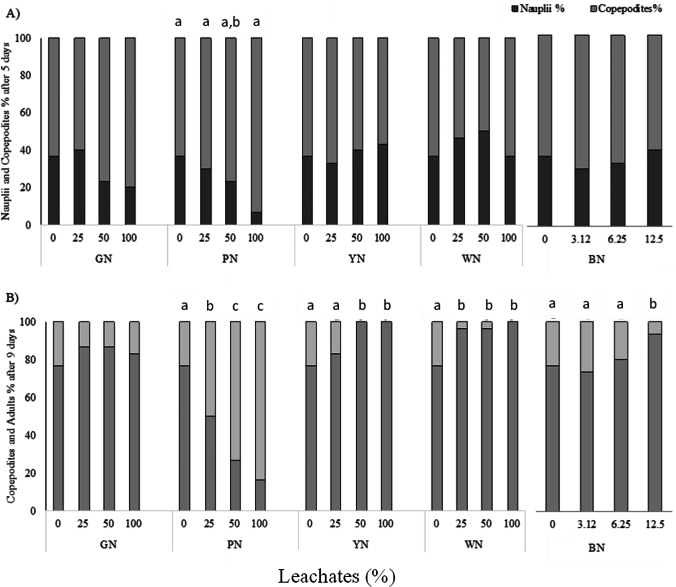
Larval development of *T. fulvus* exposed to different concentrations of GN, PN, YN, and WN leachates during sub-chronic test; bars represent nauplii/copepodites percentage (%) after 5 days (**A**) and copepodites/adults percentage after 9 days (**B**). Data with different letters are significantly different (*p* < 0.05, Tukey’s test); no letters indicated that results within the specific treatment/net colour) are not significantly different

After 5 days of sub-chronic exposure, no significant effect on the naupliar moults release was detected for YN, WN, and BN leachates, while a significant stimulatory effect (*p* < 0.05) was observed on copepods exposed to 100% PN and GN leachates with a rate increase of 8.3% and 6.9%, respectively (Fig. 4). Since the acute toxicity in most of the tested samples was found to be low or even absent (i.e. like for GN), it was possible to calculate only the EC20 values for copepodite moults reduction after 9 days of exposure. The EC20 values obtained for BN and YN MPs were 9.64 mg/L and 139 mg/L, respectively.

Exposure to PN and GN leachates resulted in a concentration-dependent increase in copepodite percentage, but a significant increase respect to the control occurred only at 100% PN leachate, where the percentage of nauplii developed to copepodites was 93.3% (*p* < 0.05) after 5 days (Fig. 4A).

During the first 9 days of exposure to leachates (Fig. 4B), significant stimulatory effects on copepodite moults release and on the development from copepodite to adult stage were detected for PN leachate dilutions (*p* < 0.05). The rate increase of copepodite moults respect to the control was of 10.8, 20.8, and 18.3% at 25, 50, and 100% of leachate, respectively (Fig. 4B). The percentage of copepodites that reached the adult stage was equal to 50% at the lowest dilution (i.e. 25% leachate) and 73.3% at the highest (*p* < 0.05).

Contrary, the exposure to YN, WN, and BN after 9 days, negatively affected mean number of copepodite moults/individual decreased significantly with increasing concentration reaching a percentage reduction respect to the control at 100% leachate of 13.3, 29.2, and 30% respectively (*p* < 0.05). The percentage of copepodites developed to adults also significantly decreased compared to the control (*p* < 0.05). The BN leachate was the most toxic (*EC*20 = 14.4%), while EC20 values obtained for WN and YN leachates were quite similar (57.2% and 64.8%).

## Discussion

Plastic pollution represents one of the main threats to aquatic ecosystems: the high production level and the slow degrading time have led to an increasing number of plastic-derived debris in oceans and seas. In this study, we assessed the potential acute and sub-lethal toxic effects of waste mussel nets, of five different colours, on copepod *T. fulvus* nauplii, both as MPs and as leachates.

To the best of our knowledge, there are few data on the toxicity of waste derelict plastics on marine organisms as most of the experiments investigated the toxicity of virgin plastic or new plastic products (Nobre et al. 2015). The characterization of the different colours of microplastic nets showed an aggregation of polypropylene particles in artificial sea water, nevertheless Tween20 surfactant use (Beiras et al. 2018; Oliviero et al. 2019). This aspect was also confirmed by the negative ζ-potential values (Table S4). How MPs can behave highly depends on the particle surface charges and on the nature of the ions in the medium that can play a decisive role in aggregation events thus influencing the uptake mechanisms and toxicity of MPs (Bergami et al. 2016; Corsi et al. 2014). In this study, the negative charge showed by all aged nets microplastic examined and the high salt concentrations in the ASW could have contributed to protect the *T. fulvus* from MP toxicity, decreasing dispersion (López-León et al. 2005). This is well reported for polystyrene nano- and microbeads in filtered natural sea water (Bergami et al. 2016; Della Torre et al. 2014; Gambardella et al. 2018, 2017; Lee et al. 2013). During the toxicity testing, the aggregation could subtract particles from the system, thus decreasing the exposure. In fact, results from this study showed that all aged mussel nets did not affect the survival of the *T. fulvus* nauplii neither at very high concentrations (150 mg/L) except for BN. Results from acute tests did not allow the calculation of LC50, except for BN nets presenting LC50 values equal to 107 mg/L and 50.1% for MPs and leachates, in that order.

These results were in accordance with other studies where concentrations from 0.001 to 10 mg/L of microplastics showed no detrimental effects (Gambardella et al. 2018; Kaposi et al. 2014). Fu et al. (2019) observed higher toxicity of polyvinyl chloride (PVC) to algae (*Chlorella vulgaris*) at 10 mg/L compared to 1000 mg/L explaining this event because of particle aggregation and sedimentation in the experimental system.

Moreover, it is known that MPs are indiscriminately ingested as “prey” and can be rapidly egested in faecal pellets (Cole et al. 2013; Gonçalves et al. 2019; Vroom et al. 2017), so the mortality can be caused by insufficient nutrition or digestion inhibition. In our case, this is unlikely considering the short-term of the considered exposure. Since there were no acute toxic effects of MPs on survival, sub-lethal responses of *T. fulvus* nauplii were checked.

The prolonged exposure to MPs did not affect the survival of *T. fulvus*. This result is in line with Cole et al. (2015) who found no impact on survival of *Calanus helgolandicus* exposed to 20 μm PS (polystyrene) beads (75 particles/mL) during 9-days of experiment. Similarly, Vroom et al. (2017) found no effect on the survival of *C. finmarchicus* females exposed to 50 particles/mL and 500 particles/mL of PS for 11 days and Xie et al. (2022) where nanoplastics at environmentally relevant levels had little effect on *T. japonicus*.

All the MPs tested after 5 days did not determine any effect on larval development of *T. fulvus*, but after 9 days, the exposure to YN (50 mg/L) and BN (20 mg/L) MPs induced a slowdown of nauplii larval development. This is like Nobre et al. (2015) that reported anomalous larval development in sea urchin embryos exposed to plastic pellets (beached and virgin plastics). Moreover, Della Torre et al. (2014) showed in *P. lividus* embryos exposed to increasing concentrations (1–50 mg/mL) of positively charged polystyrene nanoparticles a high percentage of embryos blocked at an early stage. *Dunaliella tertiolecta* and *Artemia salina* exposed to negatively charged polystyrene did not show a growth inhibition, while if exposed at positively charged polystyrene, *D. tertiolecta* showed a growth inhibition (*EC*50 = 12.97 mg/L) while in *A. salina* caused mortality after 14 days of exposure (*LC*50 = 0.83 mg/L) (Bergami et al. 2017).

About leachates, all leachates from derelict mussel nets induced development alterations in *T. fulvus* nauplii, except for GN which showed no effect. PN highlighted a stimulatory moulting effect and a premature larval development, while all other leachates showed an inhibitory effect.

The toxicity found in leachate generally was greater than observed in MPs assays, supporting the possibility of a greater effect due to the absorption of heavy metals released from plastics through the body surface, rather than through the ingestion of MPs. The heavy metals are used as additives in polymer products to increase the properties of plastics. Metals such as Zn, Pb, Cr, Co, and Cd are instead used as inorganic pigment-based colourants (Hansen et al. 2013; Massos & Turner 2017), among these, colourants that contain cadmium and lead are used for all kinds of coloured polymers, lending a coloration that goes from yellow to red. Chromium is mostly used for polymers such as PVC, polyethene, and polypropylene, whereas cobalt acetate is used to provide the blue colour. Metal stabilizers and heavy-metal pigments are not chemically bound to the polymers; therefore, they could be easily leached out (Guney and Zagury 2013, Uzairu & Gimba 2010). Wang et al. (2017) reported that most metals associated with plastics debris are derived from an inherent load, therefore it is likely that most of metals in plastic nets have already been released into the environment.

Element data were analysed according to Beiras et al. (2003) calculating the theoretical toxicity unit (TU) based on a simple additive effect model present in the leachate attributable to each investigated element (TU = [element]/LC50_element_). Unfortunately, the corresponding LC50 from the literature was available only for As, Cd, Hg, Ni, Cu, and Zn on *Tigriopus* spp.. If the sum of TU was > 1, effects could be attributable mainly to element exposure. All data and analyses were summarized in Table S5 in Supplementary Materials evidencing that none of the samples was fully characterized by the proposed approach (i.e. WN presented the highest value of the sum of TUs equal to 0.5793) suggesting that other factors contributed to the final toxicity of samples. Anyway, such analysis evidenced a clear contribution to the final toxicity of WN and YN due to Cu and Zn concentrations, while WN presented the highest TU for As, while BN for Cd. Such results only partially agree with principal component analysis evidenced in Fig. 2 suggesting that samples presented specific ecotoxicological fingerprints that could be influenced by the compounds used for their colouring (i.e. samples are from the same area and made of the same material, but different in colour) as already evidenced in Oliviero et al. (2019) using *P. lividus* as testing species.

Considering that nets utilized in this study were collected from a circumscribed sampling area from Mar Piccolo (Taranto) and they are all made of PP, additives used for colouring purposes might have contributed to alter samples’ fingerprint about metalloids and metals. Probably, metalloids and metals cannot be deemed as the only potential chemical agents responsible of the registered toxicity. Derelict plastic material can concentrate organic contaminants from the surrounding sediment or water column (Hüffer et al. 2018), especially in highly impacted industrial areas (Manfra et al. 2021) so further analyses focused on organic pollutants are necessary coupled to a specific census of the Mar Piccolo area about the existing levels of plastic debris impact. In fact, polyaromatic hydrocarbons (PAHs), polychlorobiphenyls (PCBs), hexachlorocyclohexane (HCHs), and antibiotics have been identified on microplastics with adsorbed concentrations ranging from ng/g to μg/g based on laboratory studies (Mei et al. 2020; Ziccardi et al. 2016) that can be transferred in part also to leachates during their preparation (Yang et al. 2022).

Leachates derived from different polymers varied considerably in their impacts on aquatic organisms, making the comparison not easy. Our results are consistent with previous data which showed that leachates derived from several categories of plastics caused little or no mortality depending on the polymer type, leached chemicals, and the exposed species (Bejgarn et al. 2015). Leachates derived from PP did not cause acute toxicity at the highest test concentration to *Daphnia magna* (250 g/L of plastic) (Lithner et al. 2012) and to *Nitocra spinipes* (100 g/L of plastic); however, leachates from PP became significantly more toxic to *N. spinipes* after irradiation simulating weathering (Bejgarn et al. 2015), demonstrating that toxicity of the same polymer type may also vary depending on several factors. The exposure to leachates of PVC products with different colours showed different toxicity, induced a development arrest immediately after fertilization or morphological alterations in *P. lividus* larvae (Oliviero et al. 2019).

Differences in the observed biological responses could be the result of various characteristics of the used mussel nets such as colour. The colour of MPs has been reported to affect toxicity (Chen et al. 2020) and adsorption of contaminants (Antunes et al. 2013), for example, black MPs were reported to adsorb higher contaminants than white MPs (Frias et al. 2010). Moreover, several studies revealed a positive relationship between absorption of chemicals and the length of time plastic remain in the sea because of degradation, weathering, and fouling with organic matter. All these factors can contribute to increase the active/reactive surface area and to change the surface properties allowing concentrations of chemical contaminants to increase over time via sorption and/or bioaccumulation by biofilms (Brennecke et al. 2016; Holmes et al. 2012; Rochman et al. 2013).

## Conclusions

The use of planktonic nauplii to evaluate the toxicity of microplastics is important to help understanding the impacts on critical biological processes such as larval development, which could change the structure of populations and communities.

Our results add new important information about the effects of these emerging pollutants on early life stages crustaceans, prompting the necessity to monitor coastal populations since MPs can alter generational recruitment. Acute exposures to *T. fulvus* nauplii at concentrations above those reported in the most polluted marine waters did not cause any toxic effect. In contrast, sub-lethal exposure of leachates can determine both inhibitory effect (WN, YN, and BN) or stimulatory effect on larval development (PN), or no effect at all (GN). *Tigriopus fulvus* represented an interesting biological model organism being prey and predator at the same time, thus potentially triggering bottom-up cascade impact on both the food web and the energy flow, leading to a possible change in the marine benthic community composition and structure in the long run.

## Supplementary Information

Below is the link to the electronic supplementary material.

Supplementary file1 (DOCX 482 KB)

## Data Availability

All data generated or analysed during this study are included in this published article.
