# Supplementary material for: Multi-endpoint effects of derelict tubular mussel plastic nets on Tigriopus fulvus
Source: Environ Sci Pollut Res Int. 2022 Jun 29;29(55):83554–66. doi: 10.1007/s11356-022-21569-0 (PMC9643204; doi:10.1007/s11356-022-21569-0)
Supplement: Supplementary file 1 — Supplementary file1 (DOCX 482 KB) [file 11356_2022_21569_MOESM1_ESM.docx]

**Supplementary Materials**

Figure S1. FT-IR spectra of the five net mussels: Blue Net (BN), Green Net (GN), White Net (WN) and Yellow Net (YN). Absorbance in vertical axis, IR region cm^-1^ in horizontal axis. The magnified region shows a range of the fingerprint region, where the main differences between the spectra were observed.


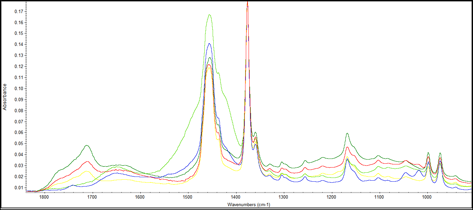


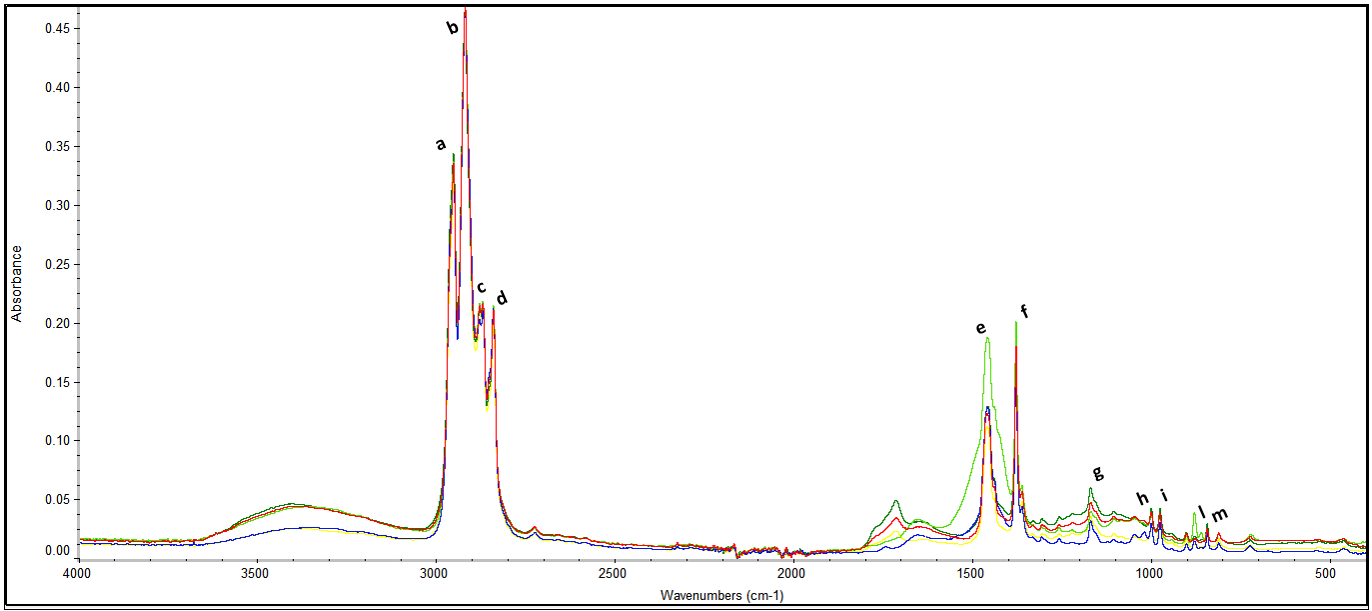


Table S1. Limit of detection (LOD) and limit of quantification (LOQ) for the detected elements.

| **Elements** | **LOD** | **LOQ** |
| --- | --- | --- |
|  | **μg L^-1^** | **μg L^-1^** |
| **Be** | 1.3 | 5 |
| **B** | 19.1 | 100 |
| **Al** | 3.2 | 10 |
| **V** | 0.9 | 5 |
| **Cr** | 0.4 | 1 |
| **Mn** | 0.2 | 1 |
| **Fe** | 1.7 | 50 |
| **Co** | 0.2 | 2 |
| **Ni** | 2.1 | 10 |
| **Cu** | 2.2 | 10 |
| **Zn** | 0.9 | 10 |
| **As** | 0.1 | 1 |
| **Mo** | 0.8 | 3 |
| **Cd** | 0.1 | 5 |
| **Sb** | 0.1 | 2 |
| **Ba** | 3.3 | 30 |
| **Hg** | 0.1 | 0.5 |
| **Pb** | 0.05 | 1 |
| **Se** | 2.4 | 10 |

Table S2. Summary of experimental conditions of acute and sub-chronic test

|  | ***Acute test*** | ***Sub-chronic test*** |
| --- | --- | --- |
| Test type | Static | Semi-static |
| Stage of development | Nauplii (≤ 24 h) | Nauplii (≤ 24 h) |
| Luminosity | 500-1200 lx cool light | 500-1200 lx cool light |
| Light/dark photoperiod | 16 h:8 h | 16 h:8 h |
| Dilution water | Artificial Sea Water IO® (0.22 µm) | Artificial Sea Water IO® (0.22 µm) |
| Salinity PSU | 38 ± 2 | 38 ± 2 |
| Temperature | 20 ± 2 | 20 ± 2 |
| pH | 8 ± 0.3 | 8 ± 0.3 |
| Chamber test | 12-well plates | 24-well plates |
| Test duration | 48 h | 5-9 d |
| Incubation volume (ml) | 3 | 1 |
| N° organisms/replicate | 10 | 1 |
| N° replicates | 3 | 10 |
| N" run | 3 | 3 |
| N° concentrations | 5 + 1 control | 3 + control |
| Solution renewal | Absent | Every 48 h |
| Feeding | Absent | *T. suecica* (1.0 x 10^5^ cell/mL) |
| Endpoint | Mortality rate | Moults number, larval development |

Table S3. Physico-chemical parameters of microplastics aged mussel nets of different colours in artificial seawater (AFW, T = 25 °C). Polydispersity index (PDI), Volume-weighted diameter (D), relative percentage of each peak (%); n.a. = not available.

|  |  | Time T= 0 |  |  |
| --- | --- | --- | --- | --- |
|  |  | Peak 1 | Peak 2 | Peak 3 |
| Mussel Nets | PDI | D (nm) ± SD |  |  |
| Pink | 0.568 | 541 ± 39 (71%) | 1.020 ± 201 (20%) | 235 ± 22 (9%) |
| White | 0.748 | 540 ± 47 (67%) | 1123 ± 121 (23%) | 184 ± 11 (10%) |
| Green | 0.678 | 533 ± 58 (67%) | 925 ± 118 (33%) | n.a. |
| Blue | 0.595 | 622 ± 38 (32%) | 1112 ± 145 (53%) | 177± 8 (15%) |
| Yellow | 0.489 | 545 ± 63 (58%) | 1234 ± 169 (34%) | 223 ± 17 (8%) |
|  |  | Time T= 1 (48h) |  |  |
|  |  | Peak 1 | Peak 2 | Peak 3 |
| Mussel Nets | PDI | D (nm) ± SD |  |  |
| Pink | 0.433 | 746 ± 118 (46%) | 1179 ± 286 (54%) | n.a. |
| White | 0.357 | 804 ± 315 (33%) | 1346 ± 362 (46%) | 367 ± 48 (22%) |
| Green | 0.646 | 947 ± 144 (100%) | n.a. | n.a. |
| Blue | 0.459 | 768 ± 142 (17%) | 1211 ± 256 (84%) | n.a. |
| Yellow | 0.324 | 676 ± 220 (68%) | 1375 ± 402 (31%) | n.a. |

Table S4. ζ-potential (mV) of aged microplastc polypropylene tested.

| Mussel nets colour | ζ-potential (mV) |
| --- | --- |
| Pink | −28.8 ± 7.5 |
| White | −37.2± 8.0 |
| Green | −25.8 ± 3.7 |
| Blue | −18.3 ± 5.7 |
| Yellow | −24.4 ± 3.9 |

Table S5 Theoretical toxicity units (TU) in elutriates attributable to each metal analysed (i.e., when the LC50 from the literature was available) according to Beiras et al. (2003)

| **Colour** | **∑TU** | TU_As* | TU_Cd* | TU_Hg** | TU_Ni** | TU_Cu** | TU_Zn** |
| --- | --- | --- | --- | --- | --- | --- | --- |
|  |  | (Forget et al. 1999) | | (Pane et al. 2008) | (Wang et al. 2020) | (Rotini et al. 2018) | (Pane et al. 2008) |
| **WN** | **0.5793** | 0.3988 | 0.0052 | 0.0001 | 0.0041 | 0.1527 | 0.0181 |
| **GN** | **0.3991** | 0.2801 | 0.0052 | 5.56E-05 | 0.0005 | 0.0989 | 0.0140 |
| **YN** | **0.3242** | 0.1005 | 0.0146 | 0.0001 | 0.0008 | 0.1565 | 0.0515 |
| **BN** | **0.3417** | 0.1010 | 0.0162 | 0.0002 | 0.0069 | 0.1291 | 0.0881 |
| **PN** | **0.3512** | 0.2336 | 0.0052 | 5.56E-05 | 0.0021 | 0.0868 | 0.0232 |
| **Tigriopus brevicornis*; ***T. fulvus* | | | | | | | |

**References**

Beiras R, Fernández N, Bellas J, Besada V, González-Quijano A, Nunes T (2003): Integrative assessment of marine pollution in Galician estuaries using sediment chemistry, mussel bioaccumulation, and embryo-larval toxicity bioassays. Chemosphere 52, 1209-24

Forget J, Pavillon J-F, Beliaeff B, Bocquené G (1999): Joint action of pollutant combinations (pesticides and metals) on survival (LC50 values) and acetylcholinesterase activity of Tigriopus brevicornis (Copepoda, Harpacticoida). Environmental Toxicology and Chemistry 18, 912-918

Pane L, Mariottini GL, Lodi A, Giacco E (2008): Effects of heavy metals on laboratory reared Tigriopus fulvus Fischer (Copepoda: Harpacticoida). Heavy metal pollution. Hauppauge, NY: Nova Science Publ, 157-65

Rotini A, Gallo A, Parlapiano I, Berducci MT, Boni R, Tosti E, Prato E, Maggi C, Cicero AM, Migliore L, Manfra L (2018): Insights into the CuO nanoparticle ecotoxicity with suitable marine model species. Ecotoxicology and Environmental Safety 147, 852-860

Wang Z, Yeung KW, Zhou G-J, Yung MM, Schlekat CE, Garman ER, Gissi F, Stauber JL, Middleton ET, Wang YYL (2020): Acute and chronic toxicity of nickel on freshwater and marine tropical aquatic organisms. Ecotoxicology and Environmental Safety 206, 111373
